# The predicting role of EFL teachers’ immediacy behaviors in students’ willingness to communicate and academic engagement

**DOI:** 10.1186/s40359-023-01378-x

**Published:** 2023-10-07

**Authors:** Li Hu, Yongliang Wang

**Affiliations:** 1 School of Foreign Languages, Chaohu University, No.1 Bantang Road, Chaohu Economic Development Zone, Hefei, Anhui 238024 China; 2 School of Foreign Languages, Nanjing Normal University, No.1 Wenyuan Road, Qixia District, Nanjing, 210046 China

**Keywords:** EFL teacher, Willingness to communicate (WTC), Teacher immediacy behaviors, Student academic engagement, SEM analysis

## Abstract

**Background:**

Teacher-student interactions and proximity have been shown influential in second/foreign (L2) education. However, the role of L2 teachers’ immediacy behaviors on students’ willingness to communicate (WTC) and academic engagement remains relatively unexamined in the context of English as a foreign language (EFL).

**Purpose:**

This study intended to examine the association among EFL teachers’ immediacy behaviors and students’ WTC and engagement.

**Methods:**

In this quantitative study, three online questionnaires were completed by 400 Chinese EFL students in different universities out of which 364 were valid.

**Results:**

The results of statistical analysis and structural equation modeling (SEM) indicated that teachers’ immediacy behaviors (verbal, nonverbal) had a highly significant influence on EFL students’ WTC (ß=0.89, p = .000) and academic engagement (ß=0.71, p = .000). It was also revealed that teachers’ immediacy could predict 89% and 71% of variances in students’ WTC academic engagement, respectively.

**Conclusions:**

Based on the results, I could be concluded that EFL teachers’ interpersonal communication abilities (e.g., immediacy) foster the establishment and growth of other positive outcomes among learners.

**Implications:**

The study presents some conclusions and practical implications for EFL teachers, materials developers, and trainers to integrate the nonverbal cues of L2 communication into their practices. Such practices have the potential to enhance students’ willingness to communicate (WTC) and improve academic engagement.

## Introduction

For a long time, the field of second/foreign language (L2) education was dominated by teaching methods that highlighted the importance and essentiality of mastering grammatical rules and vocabularies [1, 2]. However, the emergence of communicative approaches to English language teaching (ELT) shifted the attention toward communication and successful use of target language [3–5]. Communication was then regarded not only as a necessity but also as the main purpose of L2 education [6]. Nevertheless, as pinpointed in many studies, English as foreign language (EFL) students show different levels of willingness to communicate (WTC) in English, which is not their native tongue [4, 6–8]. Some L2 students willingly seek opportunities to speak, while others flee and stay silent in the classroom [6]. This sense of (un)willingness is complex and situated in a way that various factors may influence it [9]. Other than social-contextual factors, EFL students’ readiness and passion to enter into an L2 interaction at a specific time is also dependent on teacher-students’ relationships and psycho-emotional states [10–12]. The reason is that when there are positive human relations in the classroom, the students’ psyche becomes softer and their talents flourish [13].

Additionally, as widely accentuated by the proponents of positive psychology (PP), EFL teachers’ use of proper immediacy behaviors, which are communication skills, strategies, and cues, can positively influence their students’ L2 learning process [12, 14–19]. Moreover, teachers’ immediacy behaviors (verbal and non-verbal) can establish a strong sense of proximity and closeness in the classroom between the teacher and his/her students [20]. Research findings show that EFL teachers’ immediacy behaviors empower students and lead to sustained attention and increased interpersonal communication skills [21–23]. Furthermore, these behaviors can prevent and reduce negative emotions among L2 learners including their anxiety and boredom [24, 25]. Since teacher immediacy mediates among several aspects of L2 learning, such as students’ self-regulation, academic motivation and even classroom culture [26], it can influence engagement as well [15, 27–29]. Student engagement simply points to the degree of involvement in learning tasks and activities offered by the teacher/textbook [30–32]. It has a crucial role in ELT and shapes many competencies and literacies in students [33]. Given its flexibility, it is usually affected by a set of personal, phenomenological, contextual, and affective factors [34].

However, the way academic engagement and WTC of EFL students correlate, especially in light of teacher immediacy behaviors, is not clear in educational psychology research. Since the use of immediacy behaviors may establish a positive learning environment, EFL students may show more zest to interact in L2 and get immersed in their learning process. However, this interplay has been neglected by L2 scholars, so far. To fill the gaps, the present study set to examine the correlation of EFL students’ WTC and engagement with teachers’ immediacy behaviors. Moreover, it aimed to illustrate if teacher immediacy behaviors can predict the other two constructs or not. It is to contribute to a deeper understanding of the relationship between L2 learners’ emotions and interpersonal communication and teachers’ behaviors in the EFL context. Besides, by determining such a possible interplay, it exploratively constructs a structural equation modeling, which serves as a significant research guide to study teachers’ interpersonal behaviors in L2 learning process. By determining such a possible interplay, the study can enhance EFL teachers and scholars’ knowledge of L2 emotions and interpersonal communication.

## Literature review

### Teacher immediacy behaviors

In educational settings, teacher-student relations, interactions, and coexistence are crucial for an optimal performance and progress [13, 35]. These conditions are fulfilled in a positive classroom context in which teachers and learners have a strong rapport [36, 37]. Teachers’ utilization of immediacy behaviors fosters both teaching and learning [20]. Teacher immediacy behaviors refer to a spectrum of verbal and non-verbal behaviors and techniques that instructors use to constitute proximity with their pupils [20]. Basically, they are communication behaviors that demonstrate the degree of closeness among people [38, 39]. Teachers use such verbal and non-verbal cues to create a two-way communication in the classroom with students [12]. Moreover, it has been argued that teacher immediacy behaviors reduce psychological/physical distance in the class and creates a sense of liking in academia [20, 40].

The concept of immediacy is supported by the attachment theory (AT) of educational psychology, which emphasizes the importance of relational patterns and emotional ties among people [41]. According to AT, people perfume better in case they perceive a sense of bonding and belongingness to a community. Additionally, teacher immediacy is supported by PP, especially its emphasis on positive interpersonal communication behaviors. This sense of attachment to the teacher helps EFL students become relaxed, engaged, motivated, and socialized [42]. Regarding its various types, teacher immediacy behaviors can be categorized into verbal and non-verbal cues. Verbal immediacy behaviors are vocal, expressive messages, which show praise, humor, kindness, reward, empathy, inclination, and openness in classroom interactions [14]. In contrast, the goal of non-verbal cues is creating proximity between the teacher and students to enhance their participation, engagement, and attention in the class [43]. Non-verbal immediacy behaviors are associated to the use of strategies related to chronemics (time), vocalics (paralinguistic features), haptics (touch), kinesics (body movement), proxemics (distance), oculesics (eye contact), and classroom environment (arrangements) [44]. As these behaviors directly influence EFL students’ emotional involvement, they can determine the willingness and unwillingness of the learners to communicate in English, as well [45].

### Willingness to communicate in L2 education

Successful verbal communication is an indispensable part of L2 education and human relations [1, 4, 12, 46]. Although many educators may ascribe communication success to students’ communicative competence, research shows that willingness to use language authentically goes beyond linguistic issues [47]. There are many students with ordinary communicative and linguistic competence, but eager to seize every chance to interact in English, but those with a good command of English remain silent in the class [6]. This concern led to the coinage of WTC in L2 education, especially in light of MacIntyre’s scholarships. In his illuminating work, MacIntyre et al. referred to WTC as one’s zest and ability to start a probable communication when the opportunity arises [48]. The term expanded from “*unwillingness to communicate”* in first language. For years, WTC was considered a fixed personality feature. However, it is now seen as a situational construct affected by socio-cultural, attitudinal, motivational, pedagogical, and institutional factors [7]. According to MacIntyre, L2 WTC is the likelihood of initiating a conversation without terror [6]. WTC is of two types; trait and situational. Trait WTC is perceived as a continuous propensity to begin a conversation, while situational WTC arises from a particular situation [49].

**Fig. 1:**
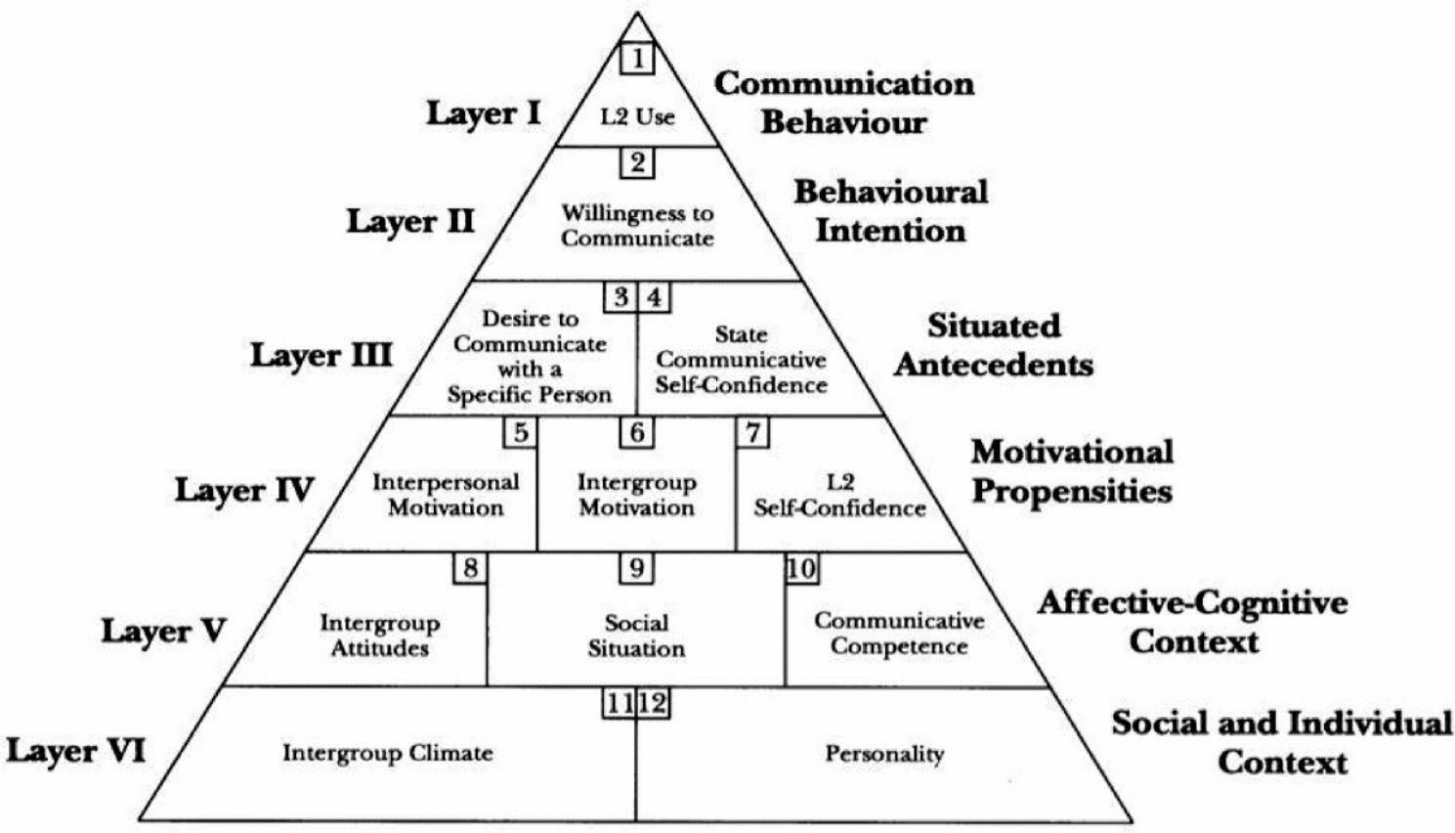
The heuristic model of WTC (MacIntyre et al., 1998, p. 547)

Concerning this variable, in their seminal model of WTC for L2 education, MacIntyre et al. argued that WTC is a complicated construct, which hinges on diverse factors related to one’s emotions, cognition, environment, and personality features [50]. Their model, which is called the Heuristic (or pyramid) model of WTC, depicts the multifaceted impact of several individual and contextual variables on every single layer on L2 communication (Fig. 1). The model is composed of six layers encompassing 12 constructs. The top layers (i.e., I, II, III) are situational and changeable, while the bottom layers (i.e., IV, V, and VI) are trait-like and more stable [51].

In light of this model, it can be argued that WTC and L2 learning are both of a multi-faceted nature and interact with different sub-systems [52]. Hence, teachers’ immediacy behaviors and students’ level of involvement in the class may determine their willingness or unwillingness to speak English.

### Student academic engagement

In the process of L2 education, students’ degree and quality of engagement in the class is vital because it guides the direction of tasks and classroom interactions [18, 53]. The concept of student engagement can be defined as learners’ concern, love, commitment, and curiosity during their learning [54]. It is associated with their cognition, participation, behavior, and feeling [55]. Engagement is a complicated and unpredictable construct in L2 education, which shows learners’ involvement in classroom tasks or activities [31]. It provides the path to academic success and shapes students’ competencies [33]. Considering its nature, the concept of student engagement, as a modern term, comprises different dimensions. It includes four facets known as cognitive, behavioral, agentic, and emotional engagement [30, 56].

As the first facet, cognitive engagement, concerns diverse mental efforts that students make to complete learning tasks/activities. Behavioral engagement has to do with how learners involve in learning tasks concerning their endeavor, awareness, participation, perseverance, and task severity. Moreover, emotional (affective) engagement alludes to students’ perceived emotions considering teachers, tasks, peers, and school [30, 33]. Lastly, agentic engagement points to students’ proactive influences on their own learning [18]. Given this multi-faceted essence, student engagement may be affected by several factors and dynamically interact with other constructs to generate overall academic success [57]. Two such constructs can be teacher immediacy and students’ WTC, which have been limitedly examined empirically.

### Previous studies

In light of shifts toward emotion-based L2 education and PP, different studies have been carried out on teachers’ immediacy behaviors [12, 20, 45, 58]. In a theoretical review, Zheng argued that teacher immediacy might correlate with clarity and credibility to foster EFL/ESL students’ engagement and motivation to learn English [23]. Additionally, Hiver et al. found that teachers’ immediacy behaviors lead to students’ motivation and self-regulation and enlighten the overall classroom culture [26]. Such behaviors can produce a cause-and-effect association with students’ emotional states [15]. Teachers’ immediacy can also promote learners’ socio-emotional development and adaptability to learning contexts [59]. It has also been found that teachers’ immediacy behaviors and cues improve their students’ attention and communication skills [21–23] and prevent their negative emotions like anxiety and boredom [24, 25]. Since communication skills and improved interpersonal interactions are tied to EFL students’ degree of WTC, it can be argued that teacher immediacy can interact with learner WTC, too [60]. In this regard, in a quantitative study in Iran, Gol et al. found a positive relationship between teacher immediacy behaviors and EFL students’ WTC [45]. They also argued that teacher immediacy is one of the underlying dimensions of L2 WTC. As shown in different studies, WTC is affected by emotional variables such as grit, enjoyment, motivation, perceptual learning style, and shyness [61–63].

Similarly, Ebn-Abbasi et al. maintained that L2 WTC has a positive correlation with students’ motivational self-systems [1]. The impact of communication-related variables like L2 communication attitude, confidence, anxiety, and self-perceived competence on L2 WTC has also been examined [8, 64, 65]. Nevertheless, the effect of teacher-related factors like immediacy behaviors on students’ WTC has remained unclear in EFL contexts. Regarding the concept of student engagement, prior research has indicated a positive correlation with factors such as motivation, agency, retention, effective learning, learning perception, resilience, ambiguity tolerance, and persistence [26, 42, 52, 66, 67]. Furthermore, it has been argued that improved academic engagement can improve learners’ socialization and well-being [68]. So far, many of the studies on EFL students’ engagement have focused on its correlation with PP constructs or its constituting dimensions. However, the way it interacts with teachers’ immediacy and L2 WTC has been neglected by researchers.

## The current study

To shed light on the role of teachers’ interpersonal factors on students’ emotions, this study sought to show the extent to which EFL teachers’ immediacy behaviors predict EFL students’ engagement and WTC is an unaddressed area of knowledge in L2 education and research. It took a quantitative approach to answer the following research question and hypotheses:

### Research question

How much variance in the EFL students’ willingness to communicate and academic engagement can be predicted by EFL teachers’ immediacy behaviors? Based on this overarching question, six research hypotheses are generated as follows:

#### NH1

Non-verbal teachers’ immediacy does not predict students’ willingness to communicate.

#### NH2

Non-verbal teachers’ immediacy does not predict students’ academic engagement.

#### NH3

Verbal teachers’ immediacy does not predict students’ willingness to communicate.

#### NH4

Verbal teachers’ immediacy does not predict students’ academic engagement.

#### NH5

Teachers’ immediacy does not predict students’ willingness to communicate.

#### NH6

Teachers’ immediacy does not predict students’ academic engagement.

## Methods

### Participants

Using convenience sampling, the researchers distributed the questionnaires among 400 Chinese EFL students. From this initial sample, 364 questionnaires were valid. This sampling technique collects data from available participants [78]. The gender of the participants was as follow: the boys 45, accounted for 12.4%; girls 319, accounted for 87.6%. The majors involved in the survey were English and Business English, and English majors had the largest proportion, accounting for 74.18%. The age range of participants is 16–30 years old, with an average age of 19–21 years. Among the students who participated in the survey, 348 were undergraduate students, accounting for 95.6%, 12 were master students, accounting for 3.3%, and 4 were doctoral students, accounting for 1.1%. The data were collected in both English and Chinese. 109 participants believed that their English proficiency is “Elementary”, accounting for 29.95%, 213 participants believed that their English proficiency is “Intermediate”, accounting for 58.52%, 40 participants chose “Upper-intermediate”, accounting for 10.99%, and another 2 participants believed that their English is “advanced”, accounting for 0.55%. They took part in the survey willingly with their formal consent form being obtained before the commencement of the research process.

### Instruments

#### Teacher immediacy behaviors questionnaire

Regarding this construct, a combination of two scales related to verbal and nonverbal immediacy behaviors of teachers was used. More specifically, the verbal and nonverbal immediacy scale of Gorham was complemented by the nonverbal immediacy measure developed by Richmond et al. [69, 70]. The verbal immediacy part included 19 items, while the nonverbal section encompassed 10 items. The items were presented on a 5-point Likert scale that ranged from 1 (never) to 5 (almost always). The reliability coefficient for this scale was estimated to be 0.85, signifying an acceptable alpha level for internal consistency.

#### Willingness to Communicate (WTC) questionnaire

In this study, MacIntyre et al.’s scale was used to measure EFL students’ WTC. The scale included 28 items asking the respondents to indicate the degree to which they might be willing to communicate in specific situations inside the classroom [71]. It was based on a 5-point Likert scale from 0 “never” to 5 “almost always”. The items of the questionnaire were divided into four skills, namely speaking (8 items), listening (5 items), reading (6 items), and writing (8 items). The internal (alpha) reliability of the scale was calculated again in this study and the results revealed an acceptable reliability of 0.79.

#### Academic Engagement questionnaire

In order to assess students’ engagement, Reeve’s questionnaire was used. It encompassed 19 items dispersed across different dimensions of academic engagement [56]. The scale was five-point Likert in which 1 represented “strongly disagree” and 5 represented “strongly agree”. The reliability of this instrument was also calculated again in the context of the study using Cronbach’s alpha. The results showed an index 0f 0.81, which is a satisfactory coefficient.

#### Data collection procedure

The data of this study were gathered through a booklet including three reliable and valid questionnaires pertaining to the variables of concern. First, the questionnaires were typed and entered into Google Forms so that an online version is created. Then, the created URL was examined and pre-viewed to detect possible typos and mistakes before collecting the main data. After checking the link, the questionnaires were distributed among 364 Chinese EFL students from different universities of Anhui and Henan provinces. They belonged to different genders, fields, and proficiency levels of English language. Before answering the items, the participants were told of the goal of the study and how to correctly answer each question. The data collection of this study lasted for 45 days and it was completed on May 29, 2023.

It is also noteworthy that this survey followed the basic research ethics, and the participants were informed of their rights and other contents that needed to be informed. Participants were aware of their rights about whether or not to participate in the study. The researchers also informed the participants that the information provided in the scales would be completely confidential and used only for research purposes. There was no previous contact and nor conflict of interests between the researchers and the respondent. All the gathered data were carefully checked and sorted out to see if they were precise and reliable. After these steps, we analyzed the data via pertinent statistical methods in light of the research question and formulated hypotheses.

### Data analysis

To analyze the data, we used different statistical techniques. First, the data were double-checked and fed into SPSS software. Then structural equation modeling (SEM) was carried out to afford a hypothetical model of the interaction of EFL teachers’ immediacy behaviors with students’ WTC and academic engagement. Afterward, goodness of fit indices was estimated for the extracted model. Subsequently, standardized regression weights were calculated for the three variables (i.e., teacher immediacy, WTC, engagement) to determine their relationship and predictive power. The final results were then demonstrated through different statistical tables and figures.

## Results

To test the model hypotheses and the research question of the study, which concerned how much variance in the EFL students’ WTC and academic engagement could be predicted by EFL teachers’ immediacy behaviors, the researchers carried out SEM analyses (Table 1).

**Table 1:** Model fit result

Model	CMIN/DF	DF	P	CMIN	GFI	NFI	CFI	RMSEA
Default model	3.204	16	0.000	112.143	0.961	0.942	0.932	0.0752
Saturated model		0		0.000	0.932	0.926	1.000	
Independence model	11.197	28	0.000	272.521	1.000	1.000	0.000	0.168

χ2 tests innately have the accompanying two significant issues practically speaking. The main issue is that T = (n − 1) F increments as n increments. Accordingly, any model structure null hypothesis will more often than not be dismissed when the example size n gets sufficiently huge, yet the model might be good enough for practical purposes. Another issue is that in SEM, the job of null and elective speculations is switched contrasted with classical hypothesis testing. Due to these deficiencies, fit files in light of test measurements have been created. These fit lists are utilized to gauge the level of in general fit of a model to data. In Table 1, the result indicated that five determiners are ratio of Cmin/df, goodness-of-fit index (GFI), normed fit index (NFI), comparative fit index (CFI), and root mean square error of approximation (RMSEA). The model fit indices are all within specifications. Therefore, Cmin/df is 3.204 (spec. ≤ 3.0), GFI = 0.961 (spec. > 0.9), NFI = 0.942 (spec. > 0.9), CFI = 0.932 (spec. > 0.9), and RMSEA = 0.075 (spec. < 0.080).

**Table 2:** Regression weights: (Group number 1 - Default model)

			Estimate	S.E.	C.R.	P	Label
Willingness to Communicate	<---	Verbal Immediacy	0.869	0.033	4.706	***	par_1
Academic Engagement	<---	Non-Verbal Immediacy	0.704	0.047	19.30	***	par_2
Academic Engagement	<---	Verbal Immediacy	0.612	0.022	9.688	***	par_3
Willingness to Communicate	<---	Non-Verbal Immediacy	0.742	0.066	-9.095	***	par_4
Willingness to Communicate	<---	Teachers’ Immediacy	0.949	0.044	21.37	***	par_5
Academic Engagement	<---	Teachers’ Immediacy	0.829	0.028	33.75	***	par_6

**Table 3:** Standardized regression weights: (Group number 1 - Default model)

			Estimate
Willingness to Communicate	<---	Verbal Immediacy	0.961
Academic Engagement	<---	Non-Verbal Immediacy	0.602
Academic Engagement	<---	Verbal Immediacy	0.578
Willingness to Communicate	<---	Non-Verbal Immediacy	0.634
Willingness to Communicate	<---	Teachers’ Immediacy	0.893
Academic Engagement	<---	Teachers’ Immediacy	0.711

The results of Tables 2 and 3 represent that the fifth null hypothesis is rejected. It means that teachers’ immediacy predicts students’ WTC. The values indicate that 89% of changes in students’ WTC can be predicted by their teachers’ immediacy. In addition, the results demonstrate that the sixth null hypothesis is rejected. In other words, 71% of changes in students’ academic engagement can be predicted by their teachers’ immediacy.

**Fig. 2:**
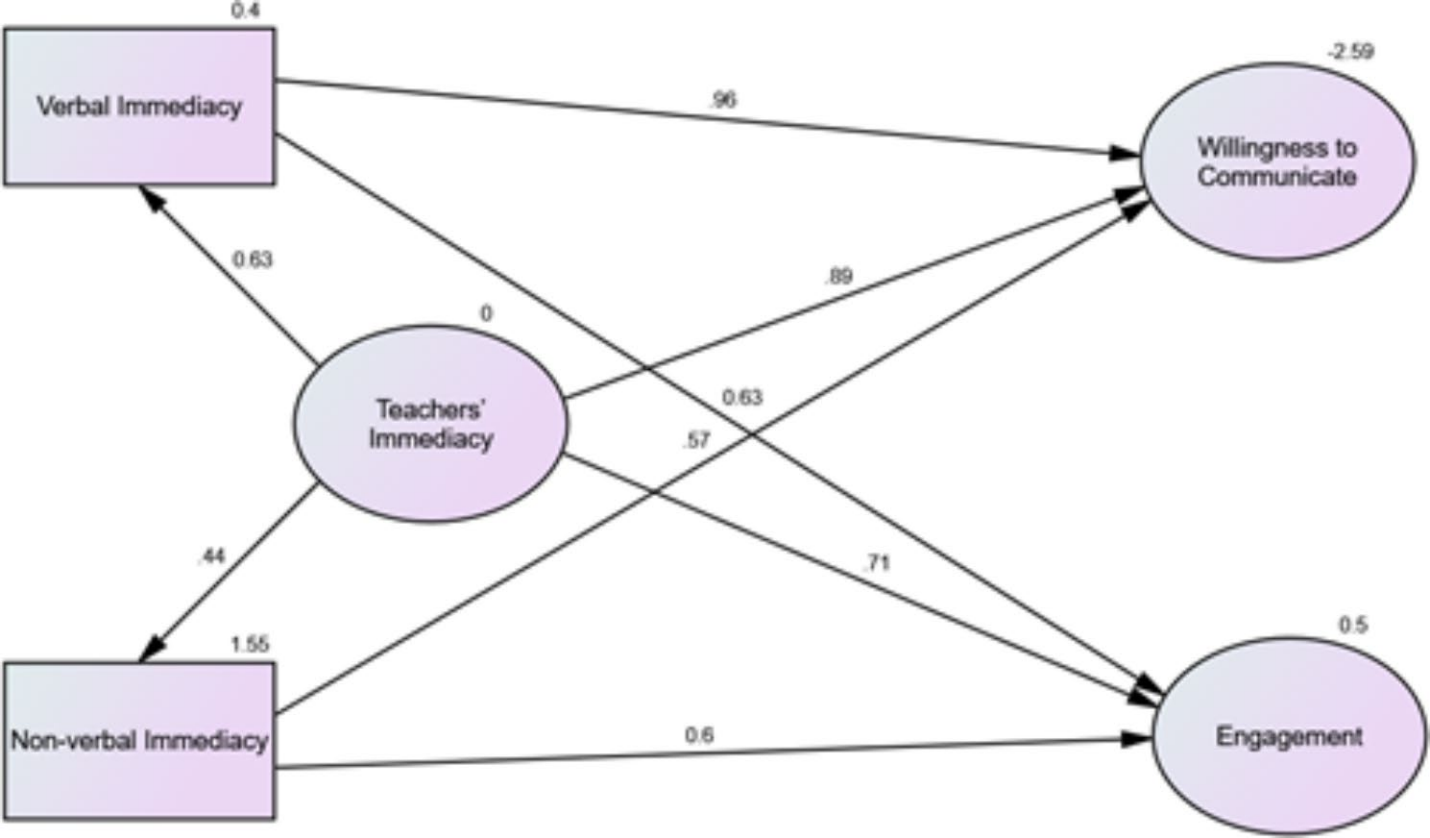
The Research Models in the standardized Estimation Mode

As has been indicated in Fig. 2, it illustrates the structural model and the obtained stable model fit estimation. The fit indices revealed Cmin/df = 3.204 (*Cmin* = 3.204, *df* = 16); GFI = 0.961 (spec. > 0.9), NFI = 0.942 (spec. > 0.9), CFI = 0.932 (spec. > 0.9), and RMSEA = 0.075 (spec. < 0.080). Concisely, Fig. 1 demonstrates that teachers’ immediacy has a highly significant impact (ß = 0.89, p = .000) on EFL students’ WTC. This figure also shows that teachers’ immediacy has a highly significant influence (ß = 0.71, p = .000) on EFL students’ academic engagement. These indicators also imply that the structural model delivered a good fit to the collected data and produced a verifying value for the good model fit. Further, the prominence of considering teachers’ immediacy in students’ WTC and their academic engagement is highlighted.

## Discussion

In this study, which examined the predictive power of Chinese EFL teachers’ immediacy behaviors in their students’ WTC and academic engagement, it was empirically found that teachers’ immediacy behaviors had a highly significant influence on EFL students’ WTC (ß=0.89, p = .000) and academic engagement (ß=0.71, p = .000). It was also illustrated that teachers’ immediacy could respectively predict 89% and71% of changes in students’ WTC and academic engagement. The results support AT, PP, and positive interpersonal communication skills in that they highlight the importance of positive emotions and interpersonal proximity in developing L2 students’ feelings as well as academic performance. In positive and friendly learning contexts, where teachers establish a close bond with learners in the class, EFL students are more likely to show academic engagement and WTC compared to a rigid setting. Empirically, this result is in accordance with those of [60], who maintained that teacher immediacy affects EFL students’ WTC in the class. The findings also confirm Gol et al.’s study, which approved the association between teacher immediacy behaviors and learners’ WTC considering immediacy as a sub-factor of L2 WTC [45]. The results also concur with Elahi Shirvan et al., Wang et al. and Zhou et al., who found that students’ academic engagement is positively affected by communication-related factors (e.g., WTC) and teacher-student interactions [8, 32, 64]. The findings can be attributed to the emotional nature of L2 education and communication. The participants considered a strong correlation among the three constructs probably because in EFL contexts, students are under affective pressures learning a foreign language. Hence, they mostly remain silent in the class until they are emotionally and linguistically prepared to initiate an L2 interaction.

The findings are also in line with Lazarides et al. and MacIntyre, who considered L2 students’ performance as the outcome of teacher-student relations and interactions [6, 13]. It is plausible that the strong emotional bond among Chinese EFL teachers and students had led the participants to consider immediacy behaviors as predictors of WTC and engagement. When there is a close sense of proximity in academia, L2 students feel more secure to initiate an L2 communication with others and be engaged in classroom activities. In contrast, when teachers are unaware of their proper use of verbal and nonverbal immediacy cues, their students may lose their attention, motivation, and involvement in the class, which, in turn, hamper their WTC. Another explanation for the findings can be the interpersonal, social, and situated basis of English language communication, which demands emotional connection between the teacher and his/her students as well as strong interpersonal communication skills [32]. It seems the participants had been familiar with the prominence of PP constructs in L2 education, especially interpersonal competences. This is attributable to their professional development training and university education.

Another justification for the observed interplay of teacher immediacy and students’ WTC and engagement could be the emphasis of the AT on relations and emotions in academia. In case teachers and students are emotionally connected, communication, social, and relational skills of the students meaningfully improve [41]. The obtained results also re-confirm the intermingled impact of teacher-psychology factors on learner-psychology factors. Many optimal outcomes related to students are the consequence of teachers’ behaviors and practices. Hence, it is logical to content that EFL teachers’ utilization of suitable verbal and nonverbal immediacy behaviors produces several outcomes on the part of students, including WTC and classroom engagement. This might be due to teachers’ high interpersonal communication skills and emotional literacy in EFL contexts. In sum, this study explicitly contributes to PP and the role of interpersonal communication skills of EFL teachers in their students’ classroom engagement and WTC.

## Conclusion and implications

The results of this study give the idea that successful L2 education and communicative competence depend not only on teachers’ pedagogical content knowledge, but also on their nonverbal-affective skills. Much of teachers’ instruction is conveyed through nonverbal signals and emotive features. Therefore, it can be concluded that EFL teachers’ knowledge of immediacy behaviors and their positive use in articulating student-talk improves language learning and communication skills among students. When the psychical-affective distance between the teacher and his/her students is kept at the minimum, L2 students feel more relaxed to take part in classroom activities and initiate conversations with others without fear. Since L2 communication can be directed by nonverbal signals, EFL teachers must be aware of such modalities that influence students’ WTC and engagement.

Saying that, the results have ramifications for EFL teachers, teacher trainers, materials developers, and syllabus designers. First, EFL teachers may use this study to deepen their understanding of the role of verbal and nonverbal uses of language in fostering students’ communication skills and zeal to involve in their learning process. Given the limitations in EFL contexts, EFL teachers can also realize the criticality of providing opportunities for their students to interact with the global community. This is achievable only through prompting students’ WTC as a pivotal element of L2 education. Second, teacher trainers may find the outcomes beneficial and run training courses in which explicit and straightforward verbal and nonverbal practices are taught to teachers to facilitate WTC and student engagement. They can also explain the role and meaning of different nonverbal signals conveyed by a teacher’s body shape, position, tone, and facial expressions. Additionally, teacher trainers can work on different ways through which EFL teachers can build a strong immediacy with learners as a starting point of many other academic outcomes.

Third, the study puts forward implications for materials developers, who can develop tasks, items, practices, and activities that minimize the distance between the teacher and his/her students. They can develop materials that improve students’ communicative ability, WTC, and engagement [72]. Likewise, materials for nonverbal cues of L2 communication can be developed via practical exercises. Finally, the results may be enlightening to syllabus designers in that they can devote emotion-based topics, activities, and assignments to each instructional session in a way that students’ WTC and academic engagement are promoted. They can also use communicative teaching methods and total physical response (TPR) principles and strategies to prompt the use of language and nonverbal cues.

Concerning the limitations of the study, it is noteworthy that the data were collected from a single context. Hence, the findings cannot be conclusively generalizable to other EFL contexts. The collected data were self-reported and this poses potential biases on the results. No comparison was made between male and female students’ perceptions regarding teacher immediacy, WTC, and engagement as gender is a crucial factor in shaping psycho-emotional constructs in L2 education. Likewise, the only source of data was an online survey and other research tools were excluded from this study. Therefore, future scholars can use mixed-methods and qualitative designs, too [73]. The correlation of teacher immediacy behaviors and other student-related constructs like resilience, optimism, grit, buoyancy, self-control, self-concept, identity etc. can be examined in the future. Future research can be done on EFL teachers and students’ perceptions about the role of teacher immediacy behaviors in different language skills. Similarly, the impact of such behaviors in the assessment performance of L2 learners, especially in alternative techniques like performance-based tests, dynamic assessment, and learning-oriented assessment are recommended [74, 75]. Additionally, the role of teachers’ immediacy behaviors in shaping and re-shaping EFL students’ assessment-related emotions can be investigated in the future, too [76, 77].

## Data Availability

The datasets used and/or analyzed during the current study are available from the first author on reasonable request.
